# 229. A Novel ‘One Health’ Approach to Understanding the Relationship of Antimicrobial Resistance Characteristics Among Humans, Bovines, and Canines

**DOI:** 10.1093/ofid/ofab466.431

**Published:** 2021-12-04

**Authors:** Laurel Legenza, John D Lee, Brooke J Olson, Song Gao, Kyle McNair, Ethan Lucas, Jessica L Hite, Thomas R Fritsche

**Affiliations:** 1 University of Wisconsin-Madison, Madison, Wisconsin; 2 Marshfield Clinic Research Institute, Marshfield, Wisconsin; 3 Marshfield Clinic Health System, Marshfield, Wisconsin

## Abstract

**Background:**

‘One Health’ recognizes the interconnectivity of humans with their production and companion animals, and the environment. Emergence and transmission of antimicrobial resistance (AMR) within and between these compartments is a recognized global threat that requires further understanding to design interventions protecting both human and animal health. In this study we identified resistance gene targets and clonotypes of *Escherichia coli* recovered from human, canine and bovine hosts and applied non-linear dimensionality reduction and visualization techniques to identify genetic relationships that may otherwise be unobservable within the data.

**Methods:**

Non-duplicative *E. coli* isolates (N=3398; see Figure captions) were collected from humans, canines, bovines from the Midwest USA. We identified beta-lactamase gene targets for third-generation cephem multidrug resistant isolates and performed clonotype analysis on each. Uniform Manifold Approximation (UMAP) was used to create a two-dimensional “map” of the high dimensional space of the genetic results to identify similarities between both infecting and colonizing isolates, and between susceptible and resistant isolates in humans and animals in the study region (see Figure captions).

**Results:**

The resulting “map” highlights similarities in: 1) genetic patterns of AMR among animals and humans, and 2) links between isolates that are infecting and colonizing in humans and canines (Figures 1-2). Our results suggest that there is strong genetic overlap linking human and animal patterns of AMR. UMAP also identified genetic segments that are unique to humans, distinct outliers, and suggest limited exchange among the neighboring counties (Figure 3).

Figure 1. Distribution of infection and surveillance isolates shows distinct clusters and distribution within host species in the UMAP space.

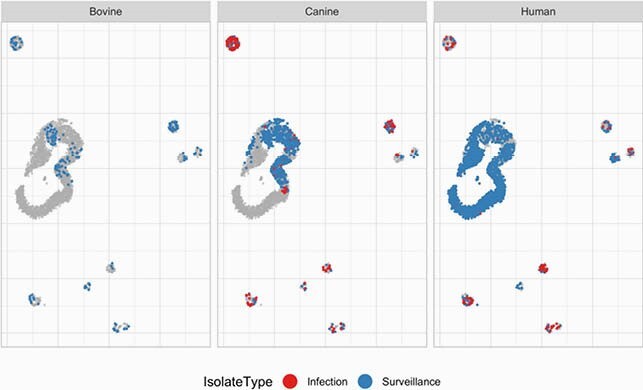

Each panel of the figure shows the same UMAP space with the labeled species in color and the other points in grey as a reference. The UMAP space is a non-linear two-dimensional representation of the genetic information contained in the clonotype analysis. UMAP is a dimensionality reduction technique similar to principal component analysis (PCA), except that it uses a non-linear combination of the underlying dimensions, which highlights the local structure and grouping of the cases. For more details see: Diaz-Papkovich, A., Anderson-Trocmé, L., & Gravel, S. (2021). A review of UMAP in population genetics. Journal of Human Genetics, 66(1), 85–91. Infection isolates: no bovine isolates tested, canine n=190, human n=115. Surveillance isolates: bovine n=175, canine n=747, human n=2171.

Figure 2. Distribution of resistant and susceptible isolates shows the resistant cases are distributed in small clusters surrounding a large cluster of predominantly susceptible cases.

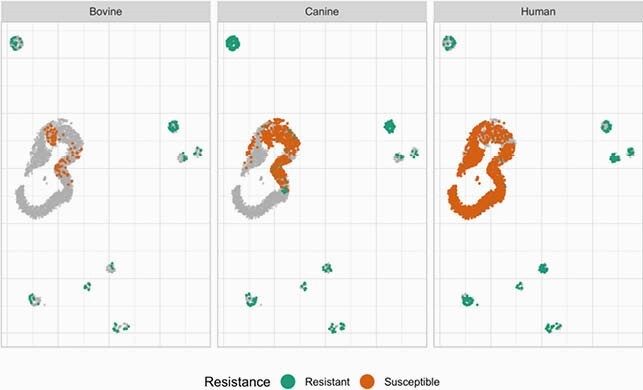

This figure plots the same cases on the same UMAP space as Figure 1. The only difference is the color that distinguishes between resistant and susceptible cases. Resistant isolates: bovine n=91, canine n=300; human n=238. Susceptible isolates: bovine n=84. canine=637, human n=2048.

Figure 3. The proportion of cases from each cluster in four adjoining counties varies considerably.

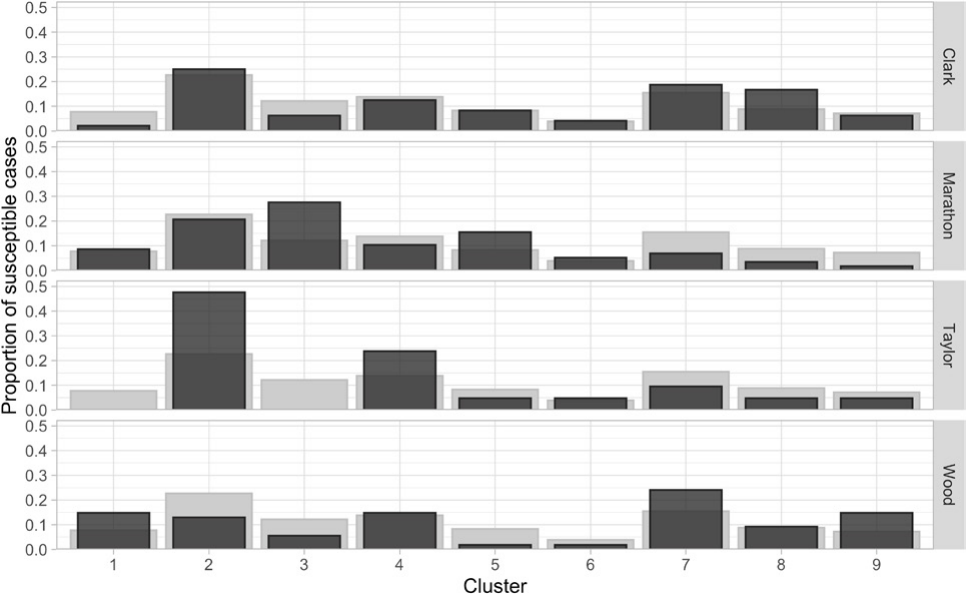

The dark bars show the proportion of cases falling into each cluster for each county. The light bars provide a reference point for interpreting the dark bars by showing the proportion of cases falling into each cluster across all four counties. When the dark bars exceed the light bars it indicates that the proportion of cases in that cluster exceeds that of the neighboring counties, such as Cluster 2 for Taylor county and Cluster 3 for Marathon county. All counties shown include a population of at least 20,000. These stipulations are in compliance with federal (HIPAA) guidelines.

**Conclusion:**

The results support that UMAP is a valuable tool for visualizing genetic AMR links across species. Human-animal transmission is likely for disparate and common clonotypes.

**Disclosures:**

**All Authors**: No reported disclosures

